# Identification of novel prognostic markers in cervical intraepithelial neoplasia using LDMAS (LOH Data Management and Analysis Software)

**DOI:** 10.1186/1471-2105-6-18

**Published:** 2005-01-26

**Authors:** Rifat A Hamoudi, Amina El-Hamidi, Ming-Qing Du

**Affiliations:** 1 Division of Molecular Histopathology, Department of Pathology, University of Cambridge, Hills Road, Cambridge, CB2 2QQ, UK

## Abstract

**Background:**

Detection of Loss of Heterozygosity (LOH) is one of the most common molecular applications in the study of human diseases, in particular cancer. The technique is commonly used to examine whether a known tumour suppressor gene is inactivated or to map unknown tumour suppressor gene(s). However, with the increasing number of samples analysed using different software, no tool is currently available to integrate and facilitate the extensive and efficient data retrieval and analyses, such as correlation of LOH data with various clinical data sets.

**Results:**

An algorithm to identify prognostic disease markers is devised and implemented as novel software called LDMAS. LDMAS is a software suite designed for data retrieval, management and integrated analysis of the clinico-pathological data and molecular results from independent databases. LDMAS is used in stratification of disease stages according to clinical stage or histological features and correlation of various clinico-pathological features with molecular findings to obtain relevant prognostic markers such as those used in predicting the outcome of cervical intraepithelial neoplasia (CIN). This approach lead to the identification of novel prognostic cervical cancer markers and extraction of useful clinical information such as correlation of Human Papilloma Virus (HPV) status with CIN lesions.

**Conclusions:**

A novel software called LDMAS is implemented and used to extract and identify prognostic disease markers. The software is used to successfully identify 4 novel prognostic markers that can be used to predict the outcome of CIN. LDMAS provides an essential platform for the extraction of useful information from large amount of data generated by LOH studies. LDMAS provides three unique and novel features for LOH analysis : (1) automatic extraction of relevant data from patient records and reports (2) correlation of LOH data with clinico-pathological data and (3) storage of complex data in flexible format. The first feature automates the creation of database of clinically relevant information from huge amount of data, the second feature extracts useful biomedical information such as prognostic markers in CIN and the third feature simplifies the statistical analyses of the data and allows non-statisticians to carry out the analysis. Additionally, LDMAS can be used to extract clinically useful markers from other diseases and interface to high throughput genotyping analysis software such as GDAS used to generate LOH data from Affymetrix^® ^GeneChip Mapping arrays.

## Background

Detection of LOH is one of the most common molecular applications in the study of human diseases, in particular cancer. It is commonly used to examine whether a known tumour suppressor gene is inactivated or to map unknown tumour suppressor gene(s). Detection of LOH not only helps in understanding the molecular mechanisms underlying the development of cancer, but also provides important information useful for disease diagnosis and prognosis. LOH detection is commonly carried out by the analysis of microsatellite markers using an automated DNA sequencer. With the raw data from the sequencer being stored in one file per lane together with corresponding clinical information and patient follow up data, each LOH study [1,2] generates hundred of files that need to be organised and related in a structured format. However, with the increasing number of samples analysed using different software, no tool is currently available to facilitate the extensive and efficient data retrieval and analyses, such as correlation of LOH data with various clinical data sets. We have developed a novel software package: LOH Data Management and Analysis Software (LDMAS) in order to satisfy these needs. LDMAS can retrieve LOH data from automated DNA sequencer platform and clinical data from any patient record system and correlate different data sets according to the user's choice. Here we present how LDMAS interfaces to Genotyper software (ABI, Foster City, CA) which is used to determine the presence of LOH, and the patient record system SunQuest (San Francisco, CA), facilitating the identification of LOH markers associated with the development of CIN [3]. CIN show variable clinical behaviour despite morphological homogeneity within each subgroup. Clinically, it is vital to distinguish CIN lesions with different behaviour and identify those likely to persist and progress despite treatment.

## Implementation

### System architecture

LDMAS package is composed of three modules:

(1) MRES (Medical Report Extractor Software) which parses patient report files, extracts the information of interest and organises it into a structured format, applicable to LDAS

(2) LDAS (LOH Data Analysis Software) which obtains LOH data from Genotyper (Applied Biosystems, California) or GDAS (Affymetrix, California) software and correlates it to clinical data obtained from MRES

(3) LDMS (LOH Data Management Software) which is used to gather patients' clinico-pathological data and extract significant relationship between the various data sets

**Figure 1:**
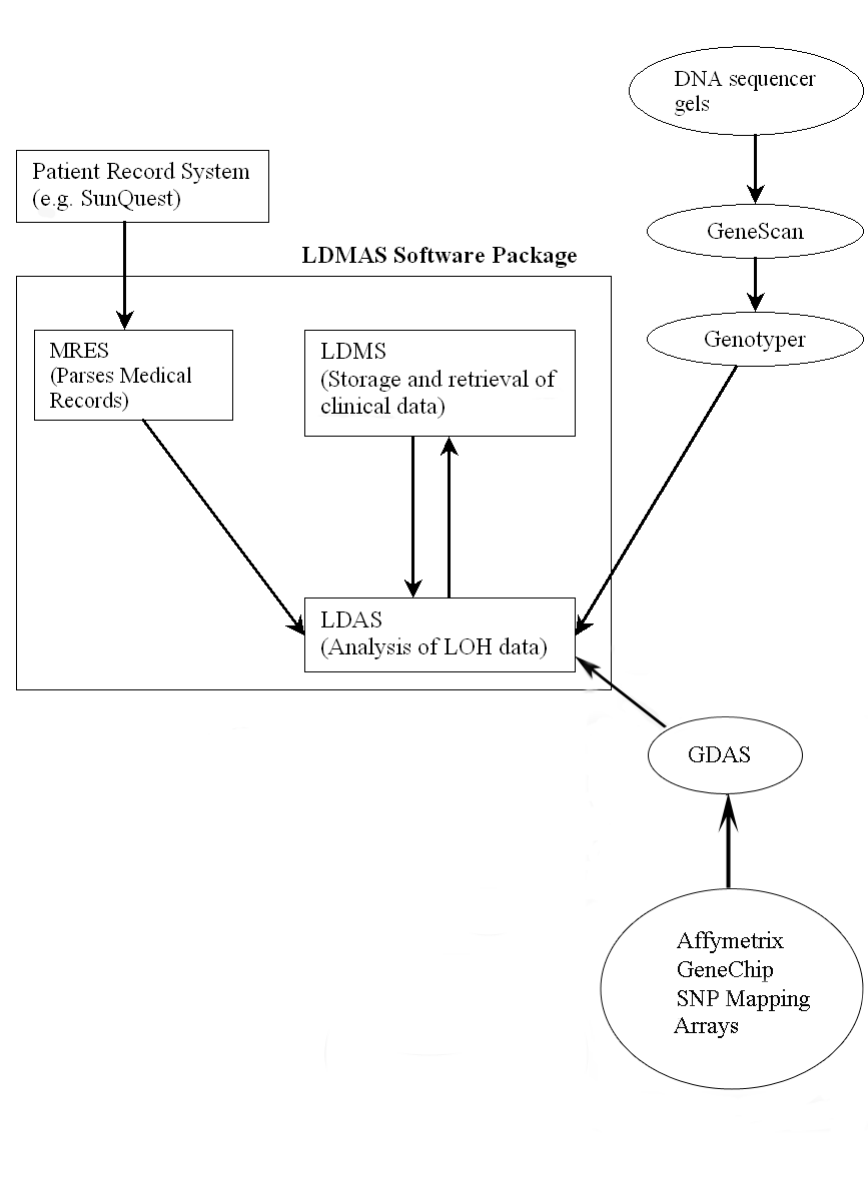
LDMAS architecture.

LDAS and LDMS work synergistically to manage and analyse LOH data. The MRES source code for automatic parsing of patient reports is written in C++ using C++ Builder 5.0 (Borland Software Corporation, Scotts Valley, CA), LDAS is written in Visual Basic for Application as an Excel 2000 add-in and LDMS is written as Visual Basic for Application modules embedded within Access 2000 as fully functional software. These modules can be run independently and used for applications other than LOH. LDMAS runs on Microsoft^® ^Windows 2000 or Windows XP operating system. Figure 1 shows LDMAS architecture.

### Type of input data for LDMAS modules

The MRES module takes its input from any patient report file containing clinical details such as diagnosis, stage of the disease, treatment and follow up results, parses and formats patient's data into a structured format that can be saved as Excel spreadsheet. In this case, data were taken from SunQuest patient record system and MRES converted and produced the data as:

(1) Hospital Number

(2) Hospital ID

(3) Patient Name

(4) Date of Birth

(5) Pathological specimen Number

(6) Date of Diagnosis

(7) Histological diagnosis.

The user can manually check the data and use it as template for analysis. Data analysis is carried out using LDAS which obtains LOH data from Genotyper and correlates it to the clinical data obtained from MRES. LDAS obtains data in plain text format and can thus be easily interfaced to any LOH platform generating software such as Genotyper and GDAS. Finally all data are entered into LDMS for storage and intelligent mining of data. Database query results can be exported back to LDAS allowing correlation between LOH and various clinico-pathological parameters such as age, histological grade, treatment modality and their responses, and HPV status as well as carry out multivariate analysis to determine the sensitivity and specificity of the markers involved in the LOH study. A more detailed description of all the modules is provided in LDMAS user's guide [Additional file 1] and the example below.

### Advantages of LDMAS

LDMAS offers several advantages to users. It is user friendly and its architecture is modular allowing versatility of use. It enforces the standardisation of procedures for studies involving large cohorts of individuals. The data is well organized since LDMAS systematically assigns LOH results of each case to its corresponding clinical information. Additionally, LDAS standardizes LOH data analysis implicitly and allows the user to edit the data manually if needed. Microsoft Excel has been chosen to implement LDAS because of its wide use, versatility and convenient statistical analysis features facilitating the implementation of multivariate analysis and correlation testing between LOH and clinico-pathological parameters.

## Results and discussion

### LDMAS application in identification of LOH markers associated with persistence / progression of cervical intraepithelial neoplasia

We divided the CIN groups into disease free indicating cases that become CIN free after treatment, and disease persistence/progression indicating cases that develop show progression or persistence of CIN despite treatment. We used LDMAS to retrospectively examine the prognostic value of LOH at 12 microsatellite markers including 10 from 3p14, 3p22-21, 6p21 and 11q23 which are frequently deleted in cervical cancer [3,4], in 164 cases of CIN lesions using archival cytological/histological specimens. LOH was further correlated with high risk HPV infection.

**Figure 2:**
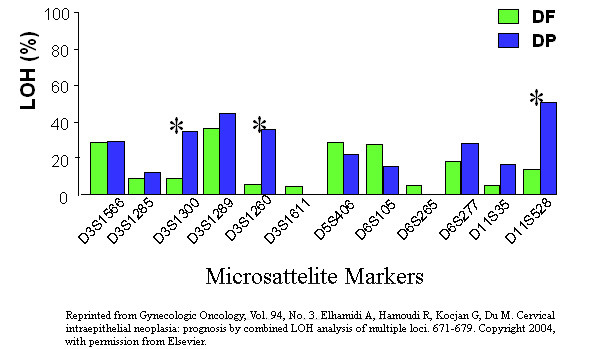
Example of output from LDMAS. LDMAS generated histogram showing the best set of LOH markers in cervical cancer highlighted by *. DF indicates Disease Free and DP indicates Disease Persistence/Progression CIN cases.

Initially MRES was used to automatically parse 4300 patient records and extract clinico-pathological data including age, diagnosis, method of treatment and treatment response during follow up. Out of those, 164 cases with follow up of 3 or more years were chosen for the study and their clinico-pathological information was imported into LDAS. Initially, 71 out of the 164 selected cases were examined for LOH using 12 fluorescent microsatellite markers ran on ABI377 DNA Sequencer. LDAS was then used to identify the microsatellite markers for which LOH was significantly associated with disease persistence/progression of CIN using two tailed student t-test. Figure 2 generated using LDAS shows that microsatellite markers D3S1300 (3p14.2), D3S1260 (3p22.2), D11S35 (11q22.1) and D11S528 (11q23.3) have the highest LOH in CIN lesions displaying persistence/progression than those who were disease free during follow up [5].

### Validation of prognostic markers associated with persistence / progression of CIN

To validate this finding, LOH at these four markers was investigated in a further series of 93 cases. Compatible results were obtained from these additional cases.

The two sets of data were combined and further compared using LDMS. Methodologies included :

1) comparison using χ^2 ^(chi-squared) test of LOH at each of the four microsatellite markers with age, various methods of treatment, different subtypes of HPV infection and between CINs showing disease free or disease persistence/progression.

2) correlation of LOH data with histological grade of CIN, treatment response and various HPV subtypes.

Through such complex analysis, we showed that concurrent LOH at two of the four microsatellite markers could identify 47% of CINs that showed disease persistence/progression with 100% specificity [5]. Furthermore, LOH at D3S1300 was found to be significantly associated with HPV16 infection. Part of this data analysis is supplied in the LDMAS guide [see Additional file 1]. More detailed analysis of this study is described in [5].

### Algorithm for identifying prognostic disease markers

Based on the above example, an algorithm can be developed to extract prognostic markers for other diseases. The algorithm can be summarised in the following pseudocode :

(1) Divide the disease in groups according to the pathology staging

(2) Parse patient data from clinical records and use the groups defined in part (1)

(3) FOR each microsatellite marker

carry out a two tailed student t-test between the disease groups using LOH data

IF t-test p ≤ 0.05

Marker is significant in prognosis of the disease

ELSE

Marker is not significant in prognosis of the disease

(4) Validate the prognostic markers using χ^2 ^(chi-squared) test of LOH with clinico-pathological data and correlation of LOH data with histological grade of CIN, treatment response and various HPV subtypes.

LDMAS has been implemented using the above pseudocode.

## Conclusions

We have devised an effective algorithm to identify and extract useful markers that can be used to predict the outcome of disease and used the algorithm to successfully identify 4 novel prognostic markers that can be used to predict the outcome of CIN. The algorithm was implemented in a novel software called LDMAS which provides an essential platform for the extraction of useful information from large amount of data generated by LOH studies. Furthermore, LDMAS is used to efficiently store, manage and track the data. Its flexible nature allows the easy manipulation of data facilitating complex analysis as demonstrated in the current study. The various modules of LDMAS can be easily adapted and used with other applications such as high throughput LOH and genotyping using SNPs on Affymetrix^® ^GeneChip Mapping arrays and fingerprinting studies. Modules such as MRES can be used independently to parse medical records facilitating extraction of specific clinical information of interest. Additionally, LDMAS can be used to extract clinically useful markers for other diseases.

## Availability and requirements

The source code and executable files for LDMAS modules as well as user manual including examples from real study data are freely available and can downloaded from our website at : 

Additionally examples of input files are provided from our website for users to test the software and assess its functionality.

## Authors' contributions

RH designed and developed and implemented LDMAS software and the web site. AE and RH did the experimental work to generate the data necessary to test and validate LDMAS. MD supervised the study and designed the experimental work using CIN biopsies and smears. All authors read and approved the final manuscript.
